# Liver transplantation for intrahepatic cholangiocarcinoma: a propensity score-matched analysis

**DOI:** 10.1038/s41598-023-37896-2

**Published:** 2023-06-30

**Authors:** Gaobo Huang, Weilun Song, Yanchao Zhang, Jiawei Yu, Yi Lv, Kang Liu

**Affiliations:** 1 National Local Joint Engineering Research Center for Precision Surgery and Regenerative Medicine, First Affiliated Hospital of Xi’an Jiaotong University, Xi’an, 710061 Shaanxi Province China; 2 Department of Oncology, Xi’an No.3 Hospital, Xi’an, 710061 Shaanxi Province China; 3 Department of Hepatobiliary Surgery, First Affiliated Hospital of Xi’an Jiaotong University, Xi’an, 710061 Shaanxi Province China

**Keywords:** Liver cancer, Liver cancer, Surgical oncology

## Abstract

Liver resection (LR) is the only recommended effective curative treatment for patients with intrahepatic cholangiocarcinoma (ICC), but the prognosis of patients with ICC is still poor even after curative resection. Recently, many researchers focused on the therapeutic value of LT for patients with ICC. This study aimed to identify the role of liver transplantation in patients with ICC by internally comparing with LR in ICC and externally comparing with LT in HCC. We obtained patient data from SEER database. Propensity score methods were applied to control confounders. Survival outcome was estimated using Kaplan–Meier survival curves and compared using the log-rank test. A total of 2538 patients with ICC after surgery and 5048 patients with HCC after LT between 2000 and 2019 were included in this study. The prognosis of patients with ICC after LT were better than patients with ICC after LR in both unmatched (HR 0.65, *P* = 0.002) and matched cohorts (HR 0.62, *P* = 0.009). The 5-year OS rate after LT could be improved to 61.7% in patients with local advanced ICC after neoadjuvant chemotherapy. In conclusion, our study demonstrated that the prognosis of patients with ICC after LT was better than patients with ICC after LR, but was still worse than patients with HCC after LT. LT with neoadjuvant chemotherapy should be considered as a treatment option for patients with locally advanced ICC, but more prospective multicenter clinical trials are needed to further confirm these results.

## Introduction

Intrahepatic cholangiocarcinoma (ICC) is the second most common liver cancer after hepatocellular carcinoma (HCC) with an increasing trend in both global incidence and mortality rate^1–3^. ICC arises from the epithelial layer of the second-degree biliary tract and has a high degree of malignancy^4,5^. Liver resection (LR) is the only effective curative treatment option for ICC^6^. However, even after curative resection, the prognosis of ICC in patients remains poor, with a 5-year overall survival (OS) rate of only 20–35%^7^.

Liver transplantation (LT), a standard treatment for early-stage HCC, can be used to treat the tumor as well as resolve underlying liver diseases, and it has shown the highest therapeutic value among all treatments available for HCC^8,9^. The 1-year and 5-year OS rates in patients with HCC after LT exceed 85% and 70%, respectively, in most centers^10^. Historically, LT has not been recommended for patients with ICC because of improper patient selection and lack of neoadjuvant therapy. However, at present, LT outcomes in patients with ICC have significantly improved due to proper patient selection and application of neoadjuvant therapies^11,12^.

Although the Milan criteria have been used to select patients with HCC to undergo LT worldwide^13,14^, to the best of our knowledge, a consensus for performing LT in patients with ICC has not yet been reached. However, two potential selection criteria have been identified: ①very early stage tumor (single tumor, tumor size ≤ 2 cm) with cirrhosis; and ②locally advanced tumor with neoadjuvant chemotherapy^11,15^.Therefore, this study aimed to identify the role of LT in patients with ICC by an internal comparison with the role of LR in patients with ICC and an external comparison with the role of LT in patients with HCC.

## Materials and methods

### Ethics statement

We analyzed data from the Surveillance, Epidemiology, and End Results (SEER) database, after signing a data agreement (11,187-Nov2021); moreover, our study was exempted from ethical review. This article does not include data obtained from human participants by any of the authors.

### Study population

We obtained patient data from the SEER Research Plus Data, 17 Registries, Nov 2021 Sub (2000–2019) incidence database, using SEER*Stat version 8.4.0. A total of 2538 patients with ICC after curative surgery and 5048 patients with HCC after LT were included in this study. The following variables were used in the analysis: patient age, sex, race, marital status, American Joint Committee on Cancer (AJCC) stage, tumor size, tumor grade, surgical approach, radiotherapy(Y/N), chemotherapy (Y/N), fibrosis score, months of survival, and OS status.

### Statistical analysis

The baseline characteristics of patients with ICC after LR and LT were compared using the Kruskal–Wallis test and χ^2^ test performed for continuous and categorical variables, respectively. To control the possible effects of the measured confounders, propensity score methods were applied. The propensity score was calculated using a multivariate logistic regression model, that included patient age, sex, race, marital status, AJCC stage, tumor size, tumor grade, radiotherapy(Y/N), chemotherapy (Y/N) and fibrosis score. Balanced cohorts were created using the one-to-one nearest-neighbor propensity score matching (PSM) method^16^. Survival was estimated using Kaplan–Meier survival curves and compared using a log-rank test.

All statistical analyses were performed using SPSS (version 24.0; SPSS, Chicago, IL, USA) and R software (version 4.1.2; http://www.r-project.org/). Statistical significance was set at *p* < 0.05.

## Results

### Patient and Tumor characteristics

A total of 2538 patients with ICC who underwent curative surgery and 5048 patients with HCC who underwent LT were enrolled in this study. Among the 2538 patients with ICC, most (95.5%) underwent LR, and only 113 (4.5%) underwent LT. Patients with ICC in the LT cohort were younger (57 vs. 65; *p* < 0.001) and had a male predilection (66.4% vs. 48.9%; *p* < 0.001) as compared to those in the LR cohort. The tumor characteristics also differed between the patients in LR and LT cohorts. Patients with an early AJCC stage, small tumor size, well-differentiated tumor grade and cirrhosis were more likely to undergo LT (*p* < 0.01). After PSM, no significant differences were observed between the patients in LR and LT cohorts. The baseline characteristics of the unmatched and matched cohorts are shown in Table 1.

**Table 1:** Demographic and tumor characteristics of patients with ICC by surgery before and after propensity score matching, SEER, 2000–2019.

Variable	Total (n = 2538)	Unmatched cohort			Propensity score-matched cohort		
		Liver resection (n = 2425)	Liver transplantation (n = 113)	*P* value	Liver resection (n = 113)	Liver transplantation (n = 113)	*P* value
Age [Median (IQR)]	65 (56–72)	65 (56–72)	57 (50–63)	< 0.001	58 (48–66)	57 (50–63)	0.891
Sex [n (%)]				< 0.001			1.000
Male	1260 (49.6%)	1185 (48.9%)	75 (66.4%)		74 (65.5%)	75 (66.4%)	
Female	1278 (50.4%)	1240 (51.1%)	38 (33.6%)		39 (34.5%)	38 (33.6%)	
Race [n (%)]				0.041			0.577
W	1998 (78.7%)	1897 (78.2%)	101 (89.4%)		103 (91.2%)	101 (89.4%)	
B	167 (6.58%)	162 (6.68%)	5 (4.42%)		7 (6.19%)	5 (4.42%)	
API	345 (13.6%)	339 (14.0%)	6 (5.31%)		3 (2.65%)	6 (5.31%)	
AI	20 (0.79%)	19 (0.78%)	1 (0.88%)		0 (0.00%)	1 (0.88%)	
Unknown	8 (0.32%)	8 (0.33%)	0 (0.00%)		0 (0.00%)	0 (0.00%)	
Marital status [n (%)]				0.547			0.560
Married	1596 (62.9%)	1527 (63.0%)	69 (61.1%)		61 (54.0%)	69 (61.1%)	
Single	849 (33.5%)	811 (33.4%)	38 (33.6%)		45 (39.8%)	38 (33.6%)	
Unknown	93 (3.66%)	87 (3.59%)	6 (5.31%)		7 (6.19%)	6 (5.31%)	
AJCC stage [n (%)]				0.001			0.982
I	830 (32.7%)	787 (32.5%)	43 (38.1%)		43 (38.1%)	43 (38.1%)	
II	418 (16.5%)	395 (16.3%)	23 (20.4%)		24 (21.2%)	23 (20.4%)	
III	647 (25.5%)	631 (26.0%)	16 (14.2%)		13 (11.5%)	16 (14.2%)	
IV	305 (12.0%)	299 (12.3%)	6 (5.31%)		27 (23.9%)	6 (5.31%)	
Unknown	338 (13.3%)	313 (12.9%)	25 (22.1%)		6 (5.31%)	25 (22.1%)	
Tumor size [n (%)]				< 0.001			0.912
0–2 cm	226 (8.90%)	199 (8.21%)	27 (23.9%)		25 (22.1%)	27 (23.9%)	
2–5 cm	862 (34.0%)	822 (33.9%)	40 (35.4%)		43 (38.1%)	40 (35.4%)	
> 5 cm	1055 (41.6%)	1048 (43.2%)	7 (6.19%)		5 (4.42%)	7 (6.19%)	
Unknown	395 (15.6%)	356 (14.7%)	39 (34.5%)		40 (35.4%)	39 (34.5%)	
Grade [n (%)]				< 0.001			0.540
I	255 (10.0%)	240 (9.90%)	15 (13.3%)		10 (8.85%)	15 (13.3%)	
II	1111 (43.8%)	1076 (44.4%)	35 (31.0%)		30 (26.5%)	35 (31.0%)	
III	670 (26.4%)	650 (26.8%)	20 (17.7%)		26 (23.0%)	20 (17.7%)	
IV	26 (1.02%)	25 (1.03%)	1 (0.88%)		3 (2.65%)	1 (0.88%)	
Unknown	476 (18.8%)	434 (17.9%)	42 (37.2%)		44 (38.9%)	42 (37.2%)	
Radiation [n (%)]				0.005			0.269
No	2121 (83.6%)	2038 (84.0%)	83 (73.5%)		91 (80.5%)	83 (73.5%)	
Yes	417 (16.4%)	387 (16.0%)	30 (26.5%)		22 (19.5%)	30 (26.5%)	
Chemotherapy [n (%)]				0.210			1.000
No	1303 (51.3%)	1252 (51.6%)	51 (45.1%)		50 (44.2%)	51 (45.1%)	
Yes	1235 (48.7%)	1173 (48.4%)	62 (54.9%)		63 (55.8%)	62 (54.9%)	
Fibrosis [n (%)]				< 0.001			0.498
Normal	354 (13.9%)	345 (14.2%)	9 (7.96%)		8 (7.08%)	9 (7.96%)	
Cirrhosis	112 (4.41%)	82 (3.38%)	30 (26.5%)		23 (20.4%)	30 (26.5%)	
Unknown	2072 (81.6%)	1998 (82.4%)	74 (65.5%)		82 (72.6%)	74 (65.5%)	

*IQR* interquartile range, *W* White, *B* Black, *AI* American Indian/Alaska Native, *API* Asian or Pacific Islander.

### Overall survival comparation between LR and LT in the ICC patients

**Figure 1:**
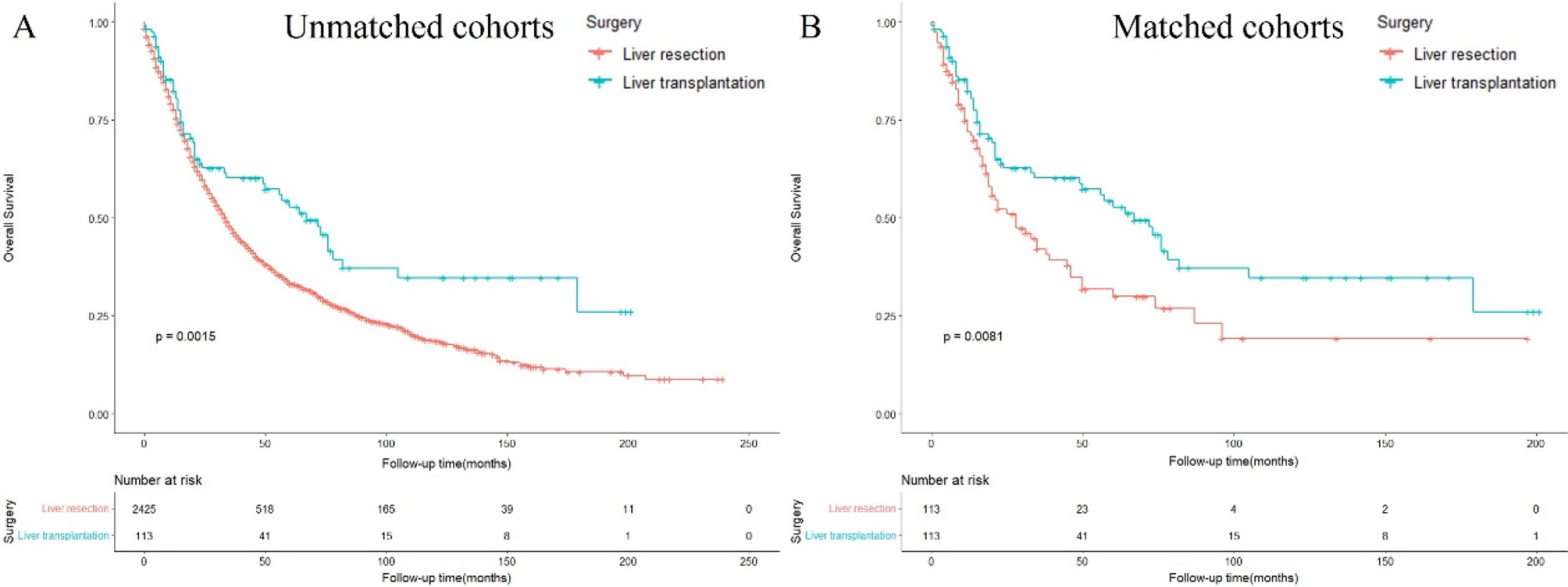
Overall survival of patients with ICC undergoing liver resection and liver transplantation in the (**A**) unmatched (**B**) matched cohorts.

In the unmatched cohorts, patients who underwent LT had significantly longer survival than those who underwent LR [median OS: 23 vs. 21 months; hazard ratio (HR): 0.65 (0.50–0.85, *p* = 0.002)] (Fig. 1A**, **Table 2). Similar outcomes were observed in patients undergoing LT and LR in the matched cohorts [median OS: 23 vs. 18 months; HR: 0.62 (0.43–0.89, *p* = 0.009)] (Fig. 1B**, **Table 2). The 5-year OS rates in patients undergoing LT and LR were 52.8% and 29.9% in the matched cohorts, respectively.

**Table 2:** OS of patients with ICC by surgery before and after propensity score matching.

	Unmatched cohort		Propensity score-matched cohort	
	Liver resection (n = 2425)	Liver transplantation (n = 113)	Liver resection (n = 113)	Liver transplantation (n = 113)
1-y OS	77.7%	82.4%	71.9%	82.4%
3-y OS	47.3%	60.3%	42.1%	60.3%
5-y OS	33.3%	52.8%	29.9%	52.8%
Median OS (months)	21 (9–44)	23 (12–69)	18 (9–39)	23 (12–69)
HR (95% CI)	Ref	0.65 (0.50–0.85, *P* = 0.002)	Ref	0.62 (0.43–0.89, *P* = 0.009)

*OS* Overall survival, *HR* Hazard ratio.

### Overall survival analysis by different selection criteria in the ICC patients receiving LT

**Figure 2:**
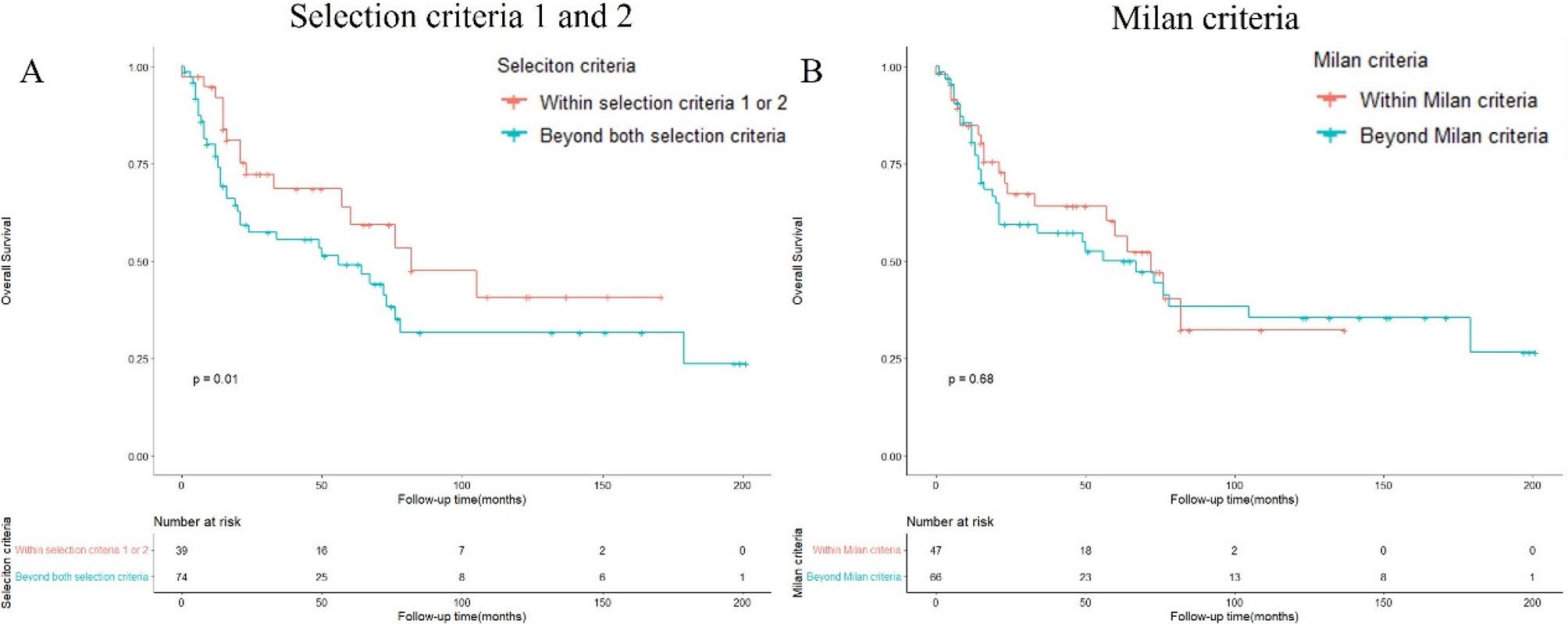
Overall survival of patients with ICC undergoing liver transplantation by selection criteria 1 and 2 (**A**) or Milan criteria (**B**).

As previously mentioned, two potential selection criteria were identified for patients with ICC undergoing LT. Herein, we defined criteria 1 as: very early stage tumor (tumor size ≤ 2 cm) + cirrhosis; and criteria 2 as: locally advanced tumor (AJCC stage I and II) + chemotherapy. After selection, 10 patients with ICC undergoing LT met the criteria 1 and 31 patients with ICC undergoing LT met the criteria 2. Survival analyses were performed for the different patient subgroups. The 5-year OS rate for patients undergoing LT who met selection criteria 1 and 2 were 43.8% and 61.7%, respectively (Table 3). The survival outcome in patients within selection criteria 1 or 2 was significantly better than patients beyond both selection criteria (*p* = 0.01) (Fig. 2A). Furthermore, we also applied Milan criteria (single tumor ≤ 5 cm or 3 tumors all ≤ 3 cm with no angioinvasion or extrahepatic involvement) to select ICC patients who underwent LT. After selection, 47 patients with ICC within Milan criteria underwent LT and 5-year OS rate of them was 56.4%. 66 patients with ICC beyond Milan criteria underwent LT and 5-year OS rate of them was 50.1% (Table 4). No significant difference in survival outcome was observed between patients within or beyond Milan criteria (*p* = 0.68) (Fig. 2B).

**Table 3:** OS of patients with ICC undergoing liver transplantation by different selection criteria.

	Liver transplantation (n = 113)			
	Beyond both selection criteria (n = 74)	Selection criteria 1 (n = 10)	Selection criteria 2 (n = 31)	Within selection criteria 1 or 2 (n = 39)
1-y OS	77.1%	90.0%	93.3%	92.1%
3-y OS	55.6%	65.6%	67.4%	68.5%
5-y OS	49.1%	43.8%	61.7%	59.4%
Median OS (months)	20 (8–66)	40 (18–55)	31(18–75)	31 (16–75)

Selection criteria 1: very early stage tumor (tumor size ≤ 2 cm) + cirrhosis, Selection criteria 2: locally advanced tumor (AJCC stage I and II) + neoadjuvant chemotherapy.

**Table 4:** OS of patients with ICC undergoing liver transplantation by Milan criteria.

	Liver transplantation (n = 113)	
	Within Milan criteria (n = 47)	Beyond Milan criteria (n = 66)
1-y OS	84.8%	80.6%
3-y OS	64.2%	57.3%
5-y OS	56.4%	50.1%
Median OS (months)	27 (15–68)	22 (12–68.5)

Milan criteria: single tumor ≤ 5 cm or 3 tumors all ≤ 3 cm with no angioinvasion or extrahepatic involvement.

### Survival analysis in patients with HCC or ICC undergoing LT

**Figure 3:**
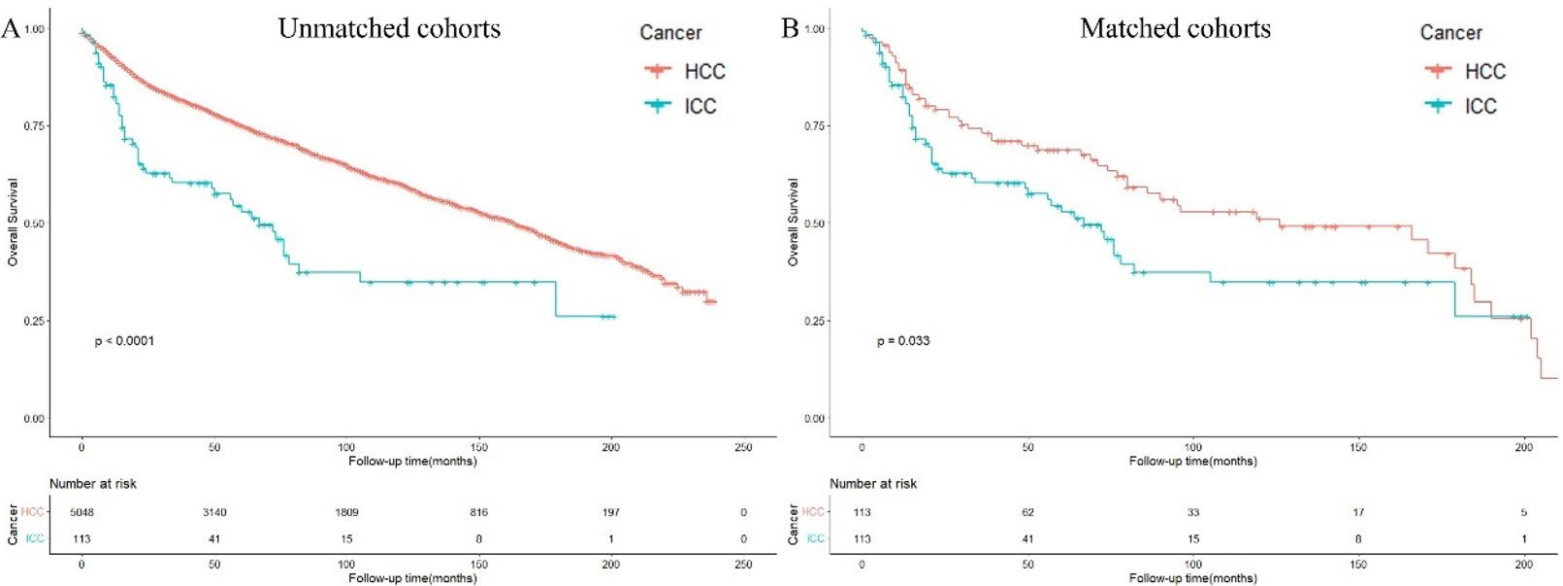
Overall survival of patients with HCC or ICC undergoing liver transplantation in the (**A**) unmatched (**B**) matched cohorts.

We further analyzed the survival outcomes of patients with ICC undergoing LT compared with those in patients with HCC. In the unmatched cohorts, patients with ICC undergoing LT had significantly shorter survival than those with HCC [median OS: 23 vs. 69 months; HR: 2.14 (1.64–2.80, *p* < 0.001)]. The 5-year OS rates in patients with ICC and patients with HCC undergoing LT were 52.8% and 74.9%, respectively (Fig. 3A, Supplementary table 1). In the matched cohorts, patients with ICC undergoing LT also had significantly worse survival outcomes than those with HCC [median OS: 23 vs. 57 months; HR: 1.52 (1.03–2.22, *p* < 0.05)] (Fig. 3B**, **Table 5).

**Table 5:** Demographic and tumor characteristics of patients with patients with HCC or ICC undergoing liver transplantation before and after propensity score matching.

Variable	Total (n = 5161)	Unmatched cohort			Propensity score-matched cohort		
		HCC (n = 5048)	ICC (n = 113)	*P* value	HCC (n = 2425)	ICC (n = 113)	*P* value
Age [Median (IQR)]	58 (52–63)	58 (52–63)	57 (50–63)	0.890	59 (52–64)	57 (50–63)	0.246
Sex [n (%)]				0.013			1.000
Male	3953 (76.6%)	3878 (76.8%)	75 (66.4%)		76 (67.3%)	75 (66.4%)	
Female	1208 (23.4%)	1170 (23.2%)	38 (33.6%)		37 (32.7%)	38 (33.6%)	
Race [n (%)]				0.066			0.912
W	4056 (78.6%)	3955 (78.3%)	101 (89.4%)		103 (91.2%)	101 (89.4%)	
B	397 (7.69%)	392 (7.77%)	5 (4.42%)		3 (2.65%)	5 (4.42%)	
API	625 (12.1%)	619 (12.3%)	6 (5.31%)		6 (5.31%)	6 (5.31%)	
AI	45 (0.87%)	44 (0.87%)	1 (0.88%)		1 (0.88%)	1 (0.88%)	
Unknown	38 (0.74%)	38 (0.75%)	0 (0.00%)		0 (0.00%)	0 (0.00%)	
Marital status [n (%)]				0.445			0.582
Married	3371 (65.3%)	3302 (65.4%)	69 (61.1%)		67 (59.3%)	69 (61.1%)	
Single	1592 (30.8%)	1554 (30.8%)	38 (33.6%)		36 (31.9%)	38 (33.6%)	
Unknown	198 (3.84%)	192 (3.80%)	6 (5.31%)		10 (8.85%)	6 (5.31%)	
Tumor size [n (%)]				< 0.001			0.863
0–2 cm	1529 (29.6%)	1502 (29.8%)	27 (23.9%)		31 (27.4%)	27 (23.9%)	
2–5 cm	2361 (45.7%)	2321 (46.0%)	40 (35.4%)		42 (37.2%)	40 (35.4%)	
> 5 cm	388 (7.52%)	381 (7.55%)	7 (6.19%)		6 (5.31%)	7 (6.19%)	
Unknown	883 (17.1%)	844 (16.7%)	39 (34.5%)		34 (30.1%)	39 (34.5%)	
Grade [n (%)]				0.001			0.776
I	1170 (22.7%)	1155 (22.9%)	15 (13.3%)		19 (16.8%)	15 (13.3%)	
II	1758 (34.1%)	1723 (34.1%)	35 (31.0%)		30 (26.5%)	35 (31.0%)	
III	389 (7.54%)	369 (7.31%)	20 (17.7%)		18 (15.9%)	20 (17.7%)	
IV	54 (1.05%)	53 (1.05%)	1 (0.88%)		0 (0.00%)	1 (0.88%)	
Unknown	1790 (34.7%)	1748 (34.6%)	42 (37.2%)		46 (40.7%)	42 (37.2%)	
Radiation [n (%)]				0.032			1.000
No	4878 (94.5%)	4795 (95.0%)	83 (73.5%)		82 (72.6%)	83 (73.5%)	
Yes	283 (5.48%)	253 (5.01%)	30 (26.5%)		31 (27.4%)	30 (26.5%)	
Chemotherapy [n (%)]				0.210			0.687
No	2864 (55.5%)	2813 (55.7%)	51 (45.1%)		47 (41.6%)	51 (45.1%)	
Yes	2297 (44.5%)	2235 (44.3%)	62 (54.9%)		66 (58.4%)	62 (54.9%)	
AFP [n (%)]				< 0.001			0.817
Normal	1400 (27.1%)	1373 (27.2%)	27 (23.9%)		31 (27.4%)	27 (23.9%)	
Elevated	1972 (38.2%)	1950 (38.6%)	22 (19.5%)		22 (19.5%)	22 (19.5%)	
Unknown	1789 (34.7%)	1725 (34.2%)	64 (56.6%)		60 (53.1%)	64 (56.6%)	
Fibrosis [n (%)]				0.142			0.914
Normal	242 (4.69%)	233 (4.62%)	9 (7.96%)		10 (8.85%)	9 (7.96%)	
Cirrhosis	1663 (32.2%)	1633 (32.3%)	30 (26.5%)		32 (28.3%)	30 (26.5%)	
Unknown	3256 (63.1%)	3182 (63.0%)	74 (65.5%)		71 (62.8%)	74 (65.5%)	

*IQR* interquartile range, *W* White, *B* Black, *AI* American Indian/Alaska Native, *API* Asian or Pacific Islander.

## Discussion

Currently, LR is the only widely accepted curative treatment for ICC. However, the 5-year OS rate in patients with ICC after LR has been reported to be 20–40%^17–19^. This was verified in our study, in which the 5-year OS rates in patients with ICC after LR were 33.3% and 29.9% in unmatched and matched cohorts, respectively. The 5-year OS rate in patients with ICC after LT was 52.8%, which was significantly higher than that in patients with ICC following LR (HR = 0.62, *p* = 0.009). The results of our study are encouraging because it is generally accepted in the transplant community that a 5-year OS rate of at least 50–60% is required for a transplant indication to be considered acceptable^20^.

In the recent years, the two potential selection criteria defined for patients with ICC undergoing LT are as follows: ①very early stage tumor (single tumor, tumor size ≤ 2 cm) with cirrhosis; and ②locally advanced tumor with neoadjuvant chemotherapy^11,15^. Our study analyzed the survival outcomes of the selected patients with ICC after LT based on these two criteria. We found that the 5-year OS rate after LT improved to 61.7% in patients with locally advanced ICC after neoadjuvant chemotherapy. However, a 5-year OS rate of 43.8% was observed in patients with very early stage ICC with cirrhosis. This unsatisfactory result could be owing to the relatively small sample size (only 10 patients with ICC had tumor size ≤ 2 cm and cirrhosis).

Regardless of the underlying disease, the goal of LT is to provide liver recipients with the maximum possible benefit from the limited donor liver source^10,21^. Thus, only the survival superiority of LT over LR or a 5-year OS rate of 61.7% did not justify LT indication in patients with ICC. Our study further analyzed the survival outcomes in patients with ICC undergoing LT compared to those in patients with HCC. However, the prognosis of ICC in patients after LT was worse than that of HCC in patients (HR: 2.14, *p* < 0.001).

Notably, the OS in the selected patients with ICC who underwent LT has significantly improved in some centers. Sapisochin et al. reported that patients with cirrhosis and very early-stage ICC (single tumor ≤ 2 cm) had good survival outcomes after LT (1-year OS: 93%, 3-year OS rate: 84% and 5-year OS rate: 65%)^22^. Lunsford et al. further reported that patients with locally advanced ICC who showed pre-transplant disease stability after neoadjuvant therapy benefited from LT. The 1-year, 3-year and 5-year OS rates were 100%, 83.3% and 83.3% respectively, although it should be noted that the sample size of this study was very small (6 patients with ICC after LT)^23^. These findings indicate the promising prospects of LT in selected patients with ICC.

## Conclusion

In conclusion, our study analyzed the survival outcomes in patients with ICC undergoing LT compared to those in patients with ICC after LR and in patients with HCC after LT. Our results demonstrated that patients with ICC after LT had a better prognosis than those after LR and the 5-year OS rate after LT was improved to 61.7% in patients with local advanced ICC after neoadjuvant chemotherapy. However, the prognosis of ICC in patients after LT was worse than that of HCC in patients after LT, which questioned the justification of performing LT in patients with ICC. LT with neoadjuvant chemotherapy should be considered as a treatment option for patients with locally advanced ICC; however, more prospective multicenter clinical trials are needed to confirm these results.

## Supplementary Information

Supplementary Table 1.

## Data Availability

Informed patient consent was not required for data obtained from SEER, as cancer is a publicly reportable disease in every state in the USA. The datasets generated and analyzed during the current study are available in SEER database. [https://seer.cancer.gov/].
