# Supplementary material for: Liver transplantation for intrahepatic cholangiocarcinoma: a propensity score-matched analysis
Source: Sci Rep. 2023 Jun 30;13:10630. doi: 10.1038/s41598-023-37896-2 (PMC10313647; doi:10.1038/s41598-023-37896-2)
Supplement: Supplementary file 1 — Supplementary Table 1. [file 41598_2023_37896_MOESM1_ESM.docx]

Supplementary table 1 OS of patients with HCC or ICC undergoing liver transplantation

|  | Liver transplantation | |
| --- | --- | --- |
|  | HCC (n=5048) | ICC (n=113) |
| 1-y OS | 92.4% | 82.4% |
| 3-y OS | 81.9% | 60.3% |
| 5-y OS | 74.9% | 52.8% |
| Median OS (months) | 69 (28-126) | 23 (12-69) |
| HR (95% CI) | Ref | 2.14 (1.64- 2.80, *P* <0.001) |

OS: Overall survival, HR: Hazard ratio.
